# Classification of Heterotic Groups and Prediction of Heterosis in Sorghum Based on Whole-Genome Resequencing

**DOI:** 10.3390/ijms26167950

**Published:** 2025-08-18

**Authors:** Hongyou Zhang, Dexin Lyu, Yu Zhang, Wei Wang, Renjie Zhao, Pengfei Lü, Wenjing Zhao, Ziyang Zhou, Shan Lu

**Affiliations:** 1 Crop Germplasm Resources Institute, Jilin Academy of Agricultural Sciences, Gongzhuling 136100, China; 2 College of Agriculture, Yanbian University, Yanbian 133002, China; 3 School of Life Science, Nanjing University, Nanjing 210023, China

**Keywords:** sorghum, whole-genome resequencing, heterotic groups, SNP markers, combining ability, heterosis prediction

## Abstract

Sorghum is a crucial food crop, and utilizing heterosis is significant for yield enhancement. To classify heterotic groups in sorghum, 96 inbred lines (48 male sterile lines and 48 restoring lines) were previously analyzed using whole-genome resequencing (WGRS) technology, from which 9691 high-quality SNP markers were obtained. In this study, the materials were divided into two groups—Group I (36 lines; predominantly restoring lines) and Group II (60 lines; mainly male sterile lines)—according to their genetic distances, and 8 lines were selected from each group for incomplete diallel crosses, producing 64 hybrid combinations for analyzing ten agronomic traits and their relationship with heterosis and combining ability. Heterosis analysis revealed that yield-related traits (plant weight, grain yield, and single-spike grain weight) exhibited the strongest heterosis, followed by morphological and developmental traits. The general combining-ability variance exceeded the specific combining-ability variance for traits controlled by additive gene effects. The results demonstrate that WGRS technology effectively classifies heterotic groups in sorghum, providing scientific support for parent selection in hybrid breeding. While combining-ability analysis offers higher predictability for heterosis than molecular genetic distance, genetic distance remains valuable for predicting heterosis.

## 1. Introduction

Sorghum (*Sorghum bicolor* Moench) originated in Africa and is the fifth-largest cereal crop in the world following wheat, barley, rice, and maize [1]. Sorghum is characterized by high photosynthetic efficiency, strong resilience, and wide adaptability, making it suitable for various applications, including brewing, natural-dye extraction, and the production of building materials [2,3]. In recent years, with the growing demand for biofuels and the intensification of climate change, sorghum has gained increasing attention as a drought-resistant and nutrient-poor-tolerant crop [4].

Heterosis refers to the phenomenon whereby hybrid offspring surpass their parents in terms of growth vigor, yield, and quality, and it is a significant pathway for increasing crop production [5]. Sorghum was one of the early crops in which heterosis was utilized, and the exploitation of heterosis has played a decisive role in significantly enhancing sorghum yields [6]. However, in practical hybrid breeding, the selection of parental lines often involves a certain degree of randomness, resulting in low breeding efficiency. Therefore, establishing effective methods for predicting heterosis is crucial for enhancing the efficiency of hybrid breeding in sorghum.

Traditional methods for predicting heterosis primarily include combining-ability analysis and the analysis of physiological and biochemical indicators [7]. With the advancement of molecular biology technologies, molecular-marker-based methods for predicting heterosis have gradually emerged [8,9]. Molecular markers can reflect genetic differences between parents at the DNA level, providing new avenues for heterosis prediction [10]. Notably, advancements in whole-genome resequencing technology have enabled the generation of high-density and high-quality single-nucleotide polymorphism (SNP) markers, providing technical support for accurate heterotic group classification and heterosis prediction [11].

Combining ability is a crucial indicator for evaluating the value of parental utilization and encompasses general combining ability (GCA) and specific combining ability (SCA) [12]. The additive effects of genes primarily determine general combining ability, which exhibits good genetic stability. In contrast, specific combining ability is influenced by the dominance effects and epistatic effects of genes and is susceptible to environmental influences [13]. The relationship between combining ability and heterosis has long been a topic of interest in breeding research [14].

Recently, we employed whole-genome resequencing technology to analyze 96 inbred lines, consisting of 48 restorers and 48 sterile lines, and generated 9691 high-quality SNP markers [15]. In this study, we conducted a genetic diversity analysis and classified the heterotic groups. We further analyzed the relationships among heterosis, combining ability, and genetic distance through incomplete diallel-cross experiments, aiming to provide a scientific basis for parental selection in sorghum hybrid breeding.

## 2. Results

### 2.1. Genetic Diversity

Genetic distances among the 96 sorghum materials were calculated based on 9691 SNP markers reported previously [15] and varied from 0.00026 to 0.01878, with an average genetic distance of 0.00767 (Table S2).

**Figure 1:**
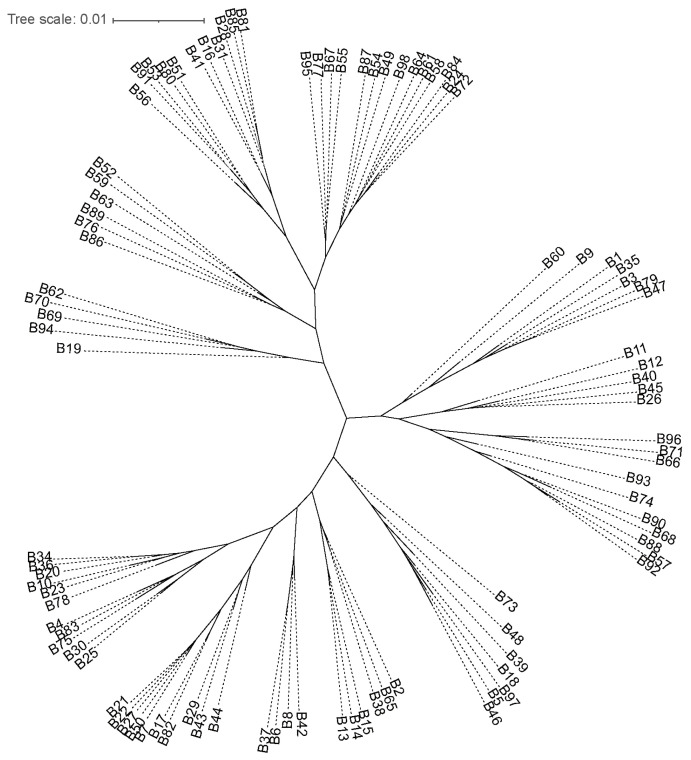
Phylogenetic tree showing the genetic relationships among samples. The unrooted tree is displayed in circular format, with branch lengths proportional to genetic distance (scale bar = 0.01 substitutions per site). Terminal branches are labeled with sample identifiers. Solid and dotted lines represent different branch types, with dotted lines potentially indicating lower support values or alternative topologies. Information for the inbred lines can be found in Table S1.

The phylogenetic tree constructed based on genetic distances divided the 96 items into two main clusters (Figure 1), which aligns closely with the results of the population structure and principal component analyses, thus validating the reliability of the clustering results. The topological structure of the phylogenetic tree indicates a high degree of genetic differentiation between the two main clusters, with a support value of 100%.

Group I comprises 36 items, consisting of 29 restorers and 7 male sterile lines, with a predominant composition of restorers. In contrast, Group II comprises 60 items, primarily consisting of maintainers, with 41 male sterile lines and 19 restorers. The results of the population structure analysis largely align with traditional classifications of male sterile lines and restorers; however, several exceptions were observed. Notably, 7 male sterile lines were assigned to Group I, while 19 restorers were classified into Group II. This discrepancy may reflect the complex genetic relationships among these materials throughout the breeding process [16].

Within Group I, the restorers were further classified into three subgroups: Subgroup I-1 comprised 4 items, which were primarily early-maturing restorers; Subgroup I-2 consisted of 19 items, which were mainly medium-maturing restorers; and Subgroup I-3 included 6 items, which were predominantly late-maturing restorers. This clustering pattern is closely related to the ecological adaptability and breeding origins of the restorers [17].

In Group II, the 41 male sterile lines also exhibited a specific clustering pattern, although the degree of differentiation was relatively lower. Notably, materials derived from the A1 cytoplasmic male sterile line were relatively clustered together, while those from the A2 cytoplasmic male sterile line formed another relatively independent branch. This observation indicates that cytoplasmic type does have an influence on the genetic relationships among the maintainers [18].

### 2.2. Heterosis Analysis

#### 2.2.1. Heterosis Performance for Various Traits

We selected eight lines from each of the two groups and produced a total of 64 hybrids. The heterosis analysis results for 10 agronomic traits across the 64 combinations indicated significant differences in heterosis expression among the different traits (Table S3). The order of mid-parent heterosis from highest to lowest was as follows: fresh biomass yield > grain yield > panicle grain weight > plant height > tiller number > ear length > thousand-grain weight > days to maturity > days from emergence to flowering > stem diameter.

The order of high-parent heterosis from strongest to weakest was as follows: tiller number > grain yield > panicle grain weight > fresh biomass yield > plant height > ear length > thousand-grain weight > days to maturity > stem diameter > days from emergence to flowering.

Traits related to yield (fresh biomass yield, grain yield, and panicle grain weight) exhibited the strongest heterosis, which aligns with the practical application of heterosis in sorghum breeding and is consistent with previous research findings [19]. Notably, the days to maturity and the number of days from emergence to flowering displayed negative heterosis, indicating that the hybrids matured earlier than their parents. This early maturity is advantageous in breeding, as it helps to mitigate risks associated with late-season drought and pest pressures [20].

The heterosis for plant height was also prominent, which is related to the C4 photosynthetic characteristics of sorghum; taller plants benefit from enhanced light-use efficiency [21]. The tiller number also showed strong heterosis, although with considerable variation, which may be attributed to environmental factors and genotype-by-environment interactions.

#### 2.2.2. Analysis of Strong Heterosis Combinations

Based on the grain yield, ten strong heterosis combinations were selected, among which ten combinations resulted from crosses between different heterosis groups (Table 1). The average heterosis index for Group I × Group II combinations is 0.67, validating the practicality of the heterosis group classification.

The most potent heterosis combination was 307A × 157, which is notable because the two parents originated from different heterosis groups with a relatively large molecular genetic distance, supporting the theory of a positive correlation between genetic distance and heterosis [22]. However, some exceptions were also observed. For instance, among the 10 weak heterosis combinations, although the QL33A × 124fu combination had the smallest genetic distance (0.0023), it still exhibited certain heterosis in some traits, which might be related to complementary effects of specific loci, suggesting that the formation mechanism of heterosis is more complex than simple genetic distance models [23].

#### 2.2.3. Genetic Basis of Heterosis

Analysis of gene frequency differences between different heterosis groups revealed that out of 9691 SNP loci, 2847 loci (29.4%) showed significant differences in gene frequency (*p* < 0.01) between the two groups. These differential loci were predominantly distributed across chromosomes 1, 2, 3, 6, and 9, indicating that these chromosomal regions may contain essential genes that affect heterosis [24].

Further analysis indicated that SNP loci within quantitative trait loci (QTL) regions associated with yield exhibited a higher degree of differentiation between the two groups, with an average FST value of 0.34, which was significantly greater than the genome-wide average (0.28). This provides insights into the molecular mechanisms underlying heterosis [25].

### 2.3. Combining Ability

The results of the variance analysis for various traits indicated that, except for grain yield, days from emergence to flowering, and days to maturity, the GCA variance for most traits was significantly greater than the SCA variance (Table 2). This suggests that additive genetic effects primarily control the heterosis observed for these traits.

Specifically, the GCA variance for plant height, ear length, and stem diameter was, respectively, 4.5, 2, and 3 times that of the SCA variance, indicating that these traits have high heritability for general combining ability, making them easier to improve in self-pollinated line breeding genetically. The GCA variances for fresh biomass yield and panicle grain weight were 2.4 and 1.2 times that of the SCA variances, suggesting that these traits are influenced by both additive and non-additive effects [17].

For grain yield, the SCA variance (55.77) was slightly greater than the GCA variance (44.23), indicating that the heterosis for yield traits is mainly determined by dominance effects and epistatic effects, which aligns with the characteristics of yield as a complex quantitative trait. In the breeding of inbred lines, the number of selection generations should be determined according to the magnitude of trait heritability values. From Table 2, it can be observed that the narrow-sense heritabilities of sorghum plant weight per plant, plant height, and thousand-grain weight were all greater than 60%, indicating that early generation selection should be applied to these traits in breeding work. The SCA variances for the days to maturity and days from emergence to flowering were 7.2 and 8.9 times the GCA variances, respectively, suggesting that the expression of these traits is significantly influenced by specific combining ability and is more susceptible to environmental conditions. The narrow-sense heritabilities of other traits were all less than 60%. Still, their broad-sense ones were all higher than 70%, indicating that these traits are significantly influenced by both additive and non-additive genetic effects and are greatly affected by environmental and cultivation conditions. Therefore, these traits might not be suitable for early generation selection. Their selection should be conducted after the materials have stabilized.

Through the analysis of GCA, it was found that among the eight sterile lines, 521A and 307A exhibited high overall GCA effect values of 7.34 and 6.11, respectively (Table 3). This indicates that these sterile lines perform well across multiple traits and can be considered core parents for breeding programs. Among the eight restoring lines, 3618 and 157 also showed high overall GCA effect values of 13.44 and 8.61, respectively.

Notably, the parent materials with high GCA effect values predominantly originated from different heterosis groups. They displayed relatively high ancestral component proportions (>85%) in the population structure analysis, suggesting a potential correlation between population structure purity and combining-ability performance [26].

Among the 64 hybrid combinations, 16 exhibited significant positive SCA effects for yield-related traits (*p* < 0.05) (Table S4). Most of these combinations resulted from crosses between different heterosis groups, and the genetic distances between the parents were relatively large on average, further validating the effectiveness of the heterosis group classification (with an average of 0.0088).

The highest SCA effect value was observed in the grain yield trait of the 170A × 3618 combination (SCA = 14.04), which also represented a combination with a significant genetic distance. This indicates that combinations of parents with higher genetic differentiation are more likely to yield strong specific combining-ability effects [27].

Among the various combinations, the specific combining abilities of 521A × 14T22, 521A × JinR7, JinchangzaoA × 307fu, and JinchangzaoA × 14T22 were predominantly positive for all traits, although the values were relatively small. The combination of 307A × JinR7 showed a positive specific combining abilities with relatively high effect values for grain yield (14.03), grains per panicle (17.44), thousand-grain weight (8.99), panicle length (11.27), plant weight per plant (10.2), tillering (39.39), and days to flowering (7.93), while plant height (−9.43), stem diameter (−1.12), and days to maturity (−1.21) showed negative specific combining abilities, indicating that this combination may produce shorter plants with longer panicles, larger grains, and higher yield, which is consistent with actual phenotypic data and aligns perfectly with current breeding objectives for modern mechanized sorghum cultivation.

Some combinations exhibited significant SCA effect values despite relatively small genetic distances. This may be associated with complementary effects at specific loci, suggesting that in parent selection, it is essential to consider not only the overall genetic distance but also the complementarity of genes related to particular traits [28].

### 2.4. Heterosis Prediction Effects

Phenotypic genetic distances were calculated (Table 4). In the analysis of phenotypic genetic distances, the maximum phenotypic genetic distance was 6.15 between QL33A and Meiza, the minimum was 2.46 between 170A and Meiza, and the average phenotypic genetic distance among parents was 4.30.

The correlation analysis between genetic distance and heterosis for various agronomic traits showed that genetic distance was positively correlated with mid-parent heterosis and high-parent heterosis for most traits. However, the correlation coefficients were generally low (Table 5). Specifically, the correlation coefficient between molecular genetic distance and mid-parent heterosis for panicle grain weight was the highest (r = 0.8; *p* < 0.01), followed by grain yield (r = 0.61; *p* < 0.05) and days to maturity (r = 0.49; *p* < 0.05). The heterosis for panicle grain weight showed a non-significant positive correlation with phenotypic genetic distance (PGD); traits such as grain yield, thousand-grain weight, panicle length, plant height, stem diameter, days to flowering, days to maturity, tillering, and plant weight per plant showed no correlation with PGD. The heterosis for grain yield, panicle grain weight, panicle length, and plant weight per plant showed highly significant positive correlations with molecular genetic distance. In contrast, traits such as plant height, stem diameter, days to flowering, days to maturity, and tillering showed no significant correlations with molecular genetic distance; however, they exhibited higher correlation coefficients with PGD, although this did not reach significance, which is consistent with the results indicating negative heterosis for these traits.

Further analysis revealed that when the genetic distance exceeded 0.01, the correlation between heterosis and genetic distance increased significantly. However, when the genetic distance was less than 0.005, the correlation was lower and even non-significant. This suggests that genetic distance can effectively predict heterosis only when a certain level of genetic differentiation exists between the parents [22].

The correlation analysis between combining ability and heterosis revealed that the correlation between GCA and heterosis was generally higher than that with molecular genetic distance (Table 6). The heterosis for grain yield, panicle grain weight, and thousand-grain weight showed significant positive correlations with GCA. Stem diameter showed a significant negative correlation with GCA, while tillering showed a highly significant negative correlation with GCA. Panicle length, plant height, and plant weight per plant were positively correlated with GCA. Days from emergence to flowering and days to maturity showed negative correlations with GCA, but these were not significant.

The heterosis for grain yield, panicle grain weight, fresh biomass yield, and plant height all showed highly significant positive correlations with SCA, while that for days from emergence to flowering showed a highly significant negative correlation with specific combining ability (SCA). In addition, the heterosis for thousand-grain weight and panicle length both showed positive but non-significant correlations with SCA, while the heterosis for stem diameter showed a negative but non-significant correlation with SCA, and the heterosis for days to maturity showed a significant negative correlation with SCA.

The correlation between specific combining ability and heterosis was closer, indicating that SCA can better predict the strength of heterosis. Particularly for yield-related traits, SCA showed significant correlations with heterosis, suggesting that combining-ability analysis has high accuracy in predicting yield heterosis.

However, combining-ability analysis requires hybrid trials, which can be costly and time-consuming. In contrast, molecular genetic distance can be assessed quickly without the need for crosses. Therefore, both methods have their advantages in breeding practices and should be selected based on specific circumstances [29].

## 3. Discussion

### 3.1. Application of Whole-Genome Resequencing in the Division of Heterosis Groups

With advancements in computational power and bioinformatics technologies, particularly the emergence of next-generation sequencing techniques, whole-genome resequencing has become efficient and cost-effective [30]. Researchers have begun to leverage genomic predictions to assist in the breeding of maize and wheat [31]. Based on an increasing body of genome sequences, whole-genome resequencing (WGRS) facilitates the selection of optimal parent lines for hybridization while reducing the costs and time associated with standard breeding cycles. Numerous WGRS studies have been reported for crop species such as rice [32], sorghum [33], tomato [34], and chickpea [35]. An important application of this technology lies in agricultural and ecological research, where it is employed to analyze genetic diversity and population structures and to enhance variety improvement.

In this study, we successfully utilized whole-genome resequencing technology to classify 96 sorghum materials into two distinct heterosis groups. This result is generally consistent with traditional classifications of sterile and restoring lines; however, some exceptions were identified. These exceptions may reflect the complex genetic relationships among the materials and offer new insights for breeding efforts.

Compared with previous studies, the range of genetic distance variation obtained in this study was relatively narrow. This may be attributed to the limited genetic basis of the materials assessed. This finding suggests that future breeding work should aim to broaden the genetic foundation by introducing materials with greater genetic diversity.

### 3.2. Comparison of Heterosis Prediction Methods

This study compared two methods for predicting heterosis: molecular genetic distance and combining ability. The results indicated that combining ability provides higher accuracy in heterosis prediction, likely due to its direct reflection of gene effects. Nevertheless, molecular genetic distance remains a crucial reference parameter in selecting parent lines due to its objective nature.

It is important to note that the mechanisms underlying heterosis are highly complex and involve multiple layers, including additive effects, dominance effects, and epistatic interactions among genes. A single prediction method often fails to capture this complexity comprehensively; therefore, developing integrated prediction models that incorporate various types of information may be a promising direction for future research.

## 4. Materials and Methods

### 4.1. Experimental Materials

The experimental materials consisted of 96 sorghum inbred lines, encompassing 48 sterile lines and 48 restoring lines, which have all been commonly used in recent years and are available from the Institute of Crop Resource institute, Jilin Academy of Agricultural Sciences, as previously reported (Table S1) [15]. Their DNA extraction, whole-genome sequencing and genetic diversity analysis were previously reported, and sequencing data are available by contacting the institute [15,36,37,38]. To assess the genetic relationship among the samples, SNP data from all samples were analyzed using the SNPhylo software (version: 20180901), which constructs a phylogenetic tree based on the Maximum Likelihood method [39]. The resulting phylogenetic tree was then visualized using the iTOL platform (https://itol.embl.de/).

### 4.2. Field Trial Design

The trials were conducted at the Gongzhuling Experimental Base of the Jilin Academy of Agricultural Sciences, China (43.50° N, 124.82° E). A randomized block design was employed with three replicates. Each plot had an area of 6 m^2^, with a row spacing of 0.65 m and a plant spacing of 0.15 m, where 8 seeds were sown per hole. Field management was carried out following conventional practices, which included weeding during the five-leaf stage and applying 20 kg of nitrogen fertilizer per acre before ear formation.

### 4.3. Hybridization Experiment Design

Eight sterile lines and eight restoring lines with greater genetic distances and significant agronomic trait differences were selected for an incomplete diallel cross, resulting in a total of 64 hybrid combinations, with 307fu used as a control.

### 4.4. Trait Measurement

Ten agronomic traits were measured at maturity, including plant height, stem diameter, ear length, panicle grain weight, thousand-grain weight, grain yield, fresh biomass yield, tiller number, days to maturity, and the number of days from emergence to flowering. Ten plants were randomly selected for measurement from each plot.

### 4.5. Data Analysis

Heterosis is a phenomenon that is not well understood but that has been exploited extensively in breeding and commercially [40]. The following formulas were used in calculations:

Mid-parent heterosis = (F1 − MP)/MP × 100%

High-parent heterosis = (F1 − HP)/HP × 100%

where F1 represents the trait value of the hybrid, MP is the mid-parent value, and HP is the highest value among the parents [41,42].

General combining ability (GCA) refers to the proportion of genotypic variance attributed to the additive effects of genes, which represents the cumulative contribution of alleles across different gene loci. In contrast, specific combining ability (SCA) denotes the portion of genotypic variance that may arise from dominance deviations, reflecting the interactions between alleles at the same locus in hybrids [43]. The method of diallel-cross analysis was employed to calculate GCA and SCA [44].

Data analysis was performed using R software (version 4.3.1), with correlation analysis conducted using Pearson correlation and significance testing performed using *t*-tests.

Fixation Index (FST) is a widely utilized genetic metric for assessing genetic differentiation among populations. FST values were computed based on population allele frequencies using the HIERFSTAT package (version 0.5-11) in R [45].

## 5. Conclusions

This study demonstrates that whole-genome resequencing technology effectively distinguishes sorghum heterosis groups, successfully classifying 96 sorghum materials into two distinct groups and thereby providing a scientific basis for parent selection in sorghum hybrid breeding. Our findings reveal significant differences in heterosis performance across various agronomic traits, with yield-related traits, including fresh biomass yield, grain yield, and panicle grain weight, exhibiting particularly strong heterosis that aligns well with practical breeding objectives. The analysis indicates that heterosis for most agronomic traits is primarily controlled by additive genetic effects, suggesting that breeding strategies for inbred line development should be tailored according to the extent of trait heritability. While combining ability demonstrates higher accuracy than molecular genetic distance in predicting heterosis, molecular genetic distance remains a vital reference parameter in parent-line selection.

## Figures and Tables

**Table 1:** Yield and special combining ability of 10 strong and weak dominant combinations.

Genotypes	Parent	GCA	SCA	PWT	Heterosis Index
157 × 307A	157	22.24	53.02	157.5	0.94
	307A	13.08			
157 × 521A	157	22.24	48.87	150	0.84
	521A	17.83			
3618 × 170A	3618	16.78	36.62	137.5	0.69
	170A	−8.91			
157 × I15A	157	22.24	6.46	137.5	0.69
	I15A	1.55			
3618 × 521A	3618	16.78	35.76	136.5	0.68
	521A	17.83			
157 × 170A	157	22.24	35.37	136.5	0.68
	170A	−8.91			
E8 × 521A	E8	−4.47	28.93	130	0.60
	521A	17.83			
3618 × 428A	3618	16.78	25.03	126	0.55
	428A	5.47			
307fu × 428A	307fu	3.86	24.41	125	0.54
	428A	5.47			
3618 × I15A	3618	16.78	23.67	124	0.52
	I15A	1.55			
124fu × JinchangzaoA	124fu	−5.17	−17.49	81.5	0.00
	JinchangzaoA	−4.79			
JinR7 × 428A	JinR7	−21.09	−27.82	73	−0.10
	428A	5.47			
14T22 × 4190A	14T22	−9.41	−25.75	72	−0.12
	4190A	−16.78			
14T22 × 170A	14T22	−9.41	−29.41	70.5	−0.13
	170A	−8.91			
JinR7 × JinchangzaoA	JinR7	−21.09	−30.24	69.5	−0.15
	JinchangzaoA	−4.79			
JinR7 × 4190A	JinR7	−21.09	−32.24	67	−0.18
	4190A	−16.78			
E8 × 170A	E8	−4.47	−38.24	60.4	−0.26
	170A	−8.91			
JinR7 × 170A	JinR7	−21.09	−40.05	60	−0.26
	170A	−8.91			
E8 × 4190A	E8	−4.47	−43.56	54	−0.34
	4190A	−16.78			
JinR7 × QL33A	JinR7	−21.09	−45.7	49.5	−0.39
	QL33A	−7.46			

**Table 2:** Genetic parameter analysis of sorghum plant-type traits.

Variance	Agronomic Traits ^1^									
	GY	PWT	TKW	PL	PH	SD	DF	DM	TL	BIY
General coordination variance%	44.23	53.67	70.88	67.19	81.79	75.02	10.13	12.23	74.63	70.45
Variance of special coordination force%	55.77	46.33	29.12	32.81	18.21	24.98	89.87	87.77	25.37	29.55
Generalized heritability%	75.22	99.38	99.05	61.55	81.69	58.21	100	99.99	48.87	98.73
Narrow heritability%	33.27	53.33	70.21	41.35	66.81	43.67	10.13	12.23	25.33	69.55

^1^ GY, grain yield (tons ha^−1^); PWT, panicle grain weight (g); TKW, thousand-kernel weight (g); PL, panicle length (cm); PH, plant height (cm); SD, stem diameter; DF, days from emergence to flowering; DM, days to maturity; TL, tiller; BIY; fresh biomass yield.

**Table 3:** General combining-ability effect of parent lines in the main agronomical traits.

Parent	Agronomic Traits ^1^										
	GY	PWT	TKW	PL	PH	SD	DF	DM	TL	BIY	Comprehensive GCA
428A	3.73	5.47	−0.9	−0.85	4.29	2.51	3.74	0.18	−0.13	3.9	2.4
521A	4.98	17.83	10.52	−4.05	12.81	0.6	2.78	−0.11	−0.2	7.71	7.34
170A	−4.26	−8.91	−9.83	−5.06	−13.31	2.3	4.86	−1.32	0.67	−15.51	−7.83
I15A	2	1.55	6.23	3.57	2.38	2.88	−5.53	−0.29	−0.79	5.81	3.15
307A	7.48	13.08	7.84	0.88	1.46	−2.51	−6.01	0.36	−0.18	16.4	6.11
JinchangzaoA	−1.73	−4.79	3.37	8.15	3.12	0.94	−2.98	0.36	−0.1	12.69	3.2
4190A	−8.59	−16.78	−13.97	1.6	−12.51	−1.04	2.46	0.46	0.31	−14.41	−9.25
QL33A	−3.61	−7.46	−3.25	−4.24	1.76	−5.69	0.7	0.36	0.31	−16.58	−5.13
E8	−2.15	−4.47	−7.72	3.1	−13.7	13.81	5.49	1.77	0.19	−1.72	−2.52
307fu	−1.57	3.86	14.23	1.34	12.37	−7.61	−6.49	0.83	−0.3	−7.83	2.57
14T22	−4.2	−9.41	−14	0.16	−16.55	3.16	−2.02	−0.29	0.33	−10.09	−7.76
157	11.65	22.24	9.07	3.48	5.06	−4.84	3.26	0.46	−0.37	16.61	8.61
3618	8.65	16.78	17.19	−10.99	54.92	−15.35	−5.21	−1.98	−0.79	12.72	13.44
124fu	−1.39	−5.17	−6.29	3.82	−3.11	0.04	−0.58	−1.7	−0.47	12.63	0.07
JinR7	−11.11	−21.09	−11.74	−1.01	−25.78	5.06	5.49	0.46	0.14	−18.98	−12.01
Meiza	0.12	−2.75	−0.74	0.11	−13.21	5.74	0.06	0.46	0.28	−3.35	−2.45

^1^ GY, grain yield (tons ha^−1^); PWT, panicle grain weight (g); TKW, thousand-kernel weight (g); PL, panicle length (cm); PH, plant height (cm); SD, stem diameter; DF, days from emergence to flowering; DM, days to maturity; TL, tiller; BIY; fresh biomass yield.

**Table 4:** Phenotypic genetic distance between parents.

Parent	428A	521A	170A	I15A	307A	JinchangzaoA	4190A	QL33A
E8	4.61	3.18	5.47	3.57	3.57	6.02	3.88	4.92
307fu	3.44	3.5	4.18	4.13	1.54	5.52	3.19	3.8
14T22	4.33	2.63	5.86	3.15	3.58	5.64	4.4	5.18
157	3.64	4.71	5.39	5.74	4.65	6.09	4.98	6.14
3618	4.04	3.7	4.61	2.84	3.18	4.1	4.49	5.86
124fu	3.79	3.76	4.39	4.13	2.63	5.86	3.03	4.6
JinR7	3.87	5.07	2.46	5	2.79	5.8	3.65	6.15
Parent	428A	521A	170A	I15A	307A	JinchangzaoA	4190A	QL33A

**Table 5:** Correlation between heterosis and genetic distance.

Genetic Distance	Agronomic Traits ^1^									
	GY	PWT	TKW	PL	PH	SD	DF	DM	TL	BIY
Phenotypic Genetic Distance	−0.34	0.66	−0.15	−0.18	0.34	−0.4	−0.07	0.46	0.53	0.42
Molecular Genetic Distance	0.61 *	0.80 **	0.4	−0.46	−0.27	−0.18	−0.29	0.43	−0.2	0.49

^1^ GY, grain yield (tons ha^−1^); PWT, panicle grain weight (g); TKW, thousand-kernel weight (g); PL, panicle length (cm); PH, plant height (cm); SD, stem diameter; DF, days from emergence to flowering; GD, DM, days to maturity; TL, tiller; BIY; fresh biomass yield. * and ** indicate the significant differences at *P* < 0.05 and 0.01 levels, respectively (Student’s *t*-test).

**Table 6:** Correlation between heterosis and combining ability ^1^.

Combining Ability	Agronomic Traits ^2^									
	GY	PWT	TKW	PL	PH	SD	DF	DM	TL	BIY
GCA	0.610 *	0.560 *	0.620 *	0.035	0.170	−0.540 *	−0.150	−0.025	−0.420	0.190
SCA	0.340 **	0.520 **	0.180	0.070	0.720 **	−0.056	−0.850 **	−0.260 *	0.310 *	0.530 **

^1^ * significance at the 5% level, ** significance at the 1% level. ^2^ GY, grain yield (tons ha^−1^); PWT, panicle grain weight (g); TKW, thousand-kernel weight (g); PL, panicle length (cm); PH, plant height (cm); SD, stem diameter; DF, days from emergence to flowering; GD, DM, days to maturity; TL, tiller; BIY; fresh biomass yield.

## Data Availability

Inquiries for original data can be directed to the corresponding authors.

## Supplementary Materials

The following supporting information can be downloaded at: https://www.mdpi.com/article/10.3390/ijms26167950/s1.

## Abbreviations

The following abbreviations are used in this manuscript:

BIY	Fresh biomass yield
DM	Days to maturity
DF	Days from emergence to flowering
GCA	General combining ability
GY	Grain yield
HP	High-parent
LD	Linkage disequilibrium
MP	Mid-parent
PGD	Phenotypic genetic distance
PH	Plant height
PL	Panicle length
PWT	Panicle weight
QTL	Quantitative trait loci
SCA	Specific combining ability
SD	Stem diameter
SNP	Single-nucleotide polymorphism
TKW	Thousand-kernel weight
TL	Tillering
WGRS	Whole-genome resequencing
